# Secondary Evolve and Resequencing: An Experimental Confirmation of Putative Selection Targets without Phenotyping

**DOI:** 10.1093/gbe/evaa036

**Published:** 2020-04-06

**Authors:** Claire Burny, Viola Nolte, Pierre Nouhaud, Marlies Dolezal, Christian Schlötterer

**Affiliations:** e1 Institut für Populationsgenetik, Vetmeduni Vienna, Austria; e2 Vienna Graduate school of Population Genetics, Vetmeduni Vienna, Austria; e3 Plattform Bioinformatik und Biostatistik, Vetmeduni Vienna, Austria

**Keywords:** experimental evolution, *Drosophila simulans*, repeatability of evolution, evolve and resequence

## Abstract

Evolve and resequencing (E&R) studies investigate the genomic responses of adaptation during experimental evolution. Because replicate populations evolve in the same controlled environment, consistent responses to selection across replicates are frequently used to identify reliable candidate regions that underlie adaptation to a new environment. However, recent work demonstrated that selection signatures can be restricted to one or a few replicate(s) only. These selection signatures frequently have weak statistical support, and given the difficulties of functional validation, additional evidence is needed before considering them as candidates for functional analysis. Here, we introduce an experimental procedure to validate candidate loci with weak or replicate-specific selection signature(s). Crossing an evolved population from a primary E&R experiment to the ancestral founder population reduces the frequency of candidate alleles that have reached a high frequency. We hypothesize that genuine selection targets will experience a repeatable frequency increase after the mixing with the ancestral founders if they are exposed to the same environment (secondary E&R experiment). Using this approach, we successfully validate two overlapping selection targets, which showed a mutually exclusive selection signature in a primary E&R experiment of *Drosophila simulans* adapting to a novel temperature regime. We conclude that secondary E&R experiments provide a reliable confirmation of selection signatures that either are not replicated or show only a low statistical significance in a primary E&R experiment unless epistatic interactions predominate. Such experiments are particularly helpful to prioritize candidate loci for time-consuming functional follow-up investigations.

## Introduction

Experimental evolution provides the opportunity to study evolutionary processes over time scales short enough to be followed experimentally (Garland and Rose 2009; Kawecki et al. 2012). The combination of high-throughput sequencing with experimental evolution (evolve and resequence) has been widely used to identify adaptive alleles across multiple replicates starting from the same reservoir of standing variation in highly similar, well-controlled environments (Turner et al. 2011; Long et al. 2015; Schlötterer et al. 2015). Evolve and resequencing (E&R) studies successfully characterized the genomic responses during adaptation to novel selective pressures usually on organisms with short generation times (e.g., Turner and Miller 2012; Burke et al. 2014; Lenski 2017; Papkou et al. 2019; Remigi et al. 2019). Laboratory natural selection experiments using the E&R framework studied responses to thermal (Orozco-terWengel et al. 2012; Tobler et al. 2014; Michalak et al. 2019) or desiccation stress (Schou et al. 2014), starvation (Michalak et al. 2019), and salt- and cadmium-enriched environments (Huang et al. 2014). The advantage of E&R studies starting from natural variation is that adaptation is possible without de novo mutations (Teotónio et al. 2009). Hence, even organisms with moderate experimental population sizes, such as *Drosophila*, are able to adapt to novel conditions within experimentally feasible time scales. Furthermore, when the starting variation is sampled from a natural population, E&R studies provide direct information about the frequency of the selected alleles in the wild (Barghi et al. 2019).

Standard statistical tests applied to E&R data (e.g., Cochran–Mantel–Haenszel [CMH] test [Agresti 2002; Spitzer et al. 2019] or Generalized Linear Modeling [Phillips et al. 2018]) require parallel selection responses across replicates. Two different, not mutually exclusive, factors can severely compromise the detection of selection targets based on these approaches. Polygenic adaptation to a new trait optimum results in reduced genomic parallelism across replicates relative to adaptation based on a few alleles of large effect (Franssen et al. 2017; Barghi et al. 2019). Furthermore, selected alleles with low starting frequencies are not only less likely to reach a detectable selection signature but genetic drifts, that is, chances, also result in lower repeatability across replicates (Lenormand et al. 2016). One further complication for the identification of selection targets with low starting frequencies arises from hitchhiking of single-nucleotide polymorphisms (SNPs) shared with haplotypes carrying the favorable allele (Nuzhdin and Turner 2013; Tobler et al. 2014; Franssen et al. 2015). In this case, the limited number of recombination events during the experiment results in large genomic regions with selection signatures when selection operates on low frequency alleles that make the identification of individual candidate genes impossible.

The functional characterization of selected alleles in E&R studies is an important next step for a better understanding of adaptation processes, but despite the recent advances based on the CRISPR/Cas9 technology (Bassett et al. 2013), the functional characterization of different alleles in a standardized genetic background is still a challenging and time-consuming task. This implies that investigators are well advised to have high confidence in alleles that are going to be functionally tested.

We propose a simple experimental procedure to validate candidate regions with weak statistical support, due to either a weak selection signature across replicates or replicate-specific selection signatures. The basic idea of this approach is that an evolved population is “diluted” with ancestral genotypes. This reduces the frequency of putatively selected alleles and the reproducible increase in frequency of selected alleles in multiple replicates evolving under the same selection regime (secondary E&R) serves as a validation of candidate regions. Because secondary E&R experiments provide the opportunity for additional recombination events, we also evaluated whether this approach increases the mapping resolution, which is particularly important for low frequency beneficial alleles.

Applying secondary E&R to a candidate region identified in *Drosophila* *simulans* populations that have been exposed to a novel constant hot environment at 23 °C for 70 generations, we demonstrate that candidate selection targets can be experimentally confirmed.

## Materials and Methods

### The Primary E&R Experiment

#### Experimental Population and Selection Regime

We collected a natural *D. simulans* population 10 km North of Stellenbosch, South Africa, in February and March 2013 and established isofemale lines that were maintained in the laboratory for approximately eight generations. For starting the primary E&R experiment, 3 mated females from each of 426 isofemale lines were combined 3 times to generate 3 replicates of the ancestral population (replicates *x*, *y*, and *z*) in F0. They were subsequently maintained as independent populations with a census population size of 1,250 and nonoverlapping generations under a constant 23 °C temperature regime with a 12-h light/12-h dark cycle (LD 12:12) for 70 generations (F70). The 426 lines used for constituting the ancestral population were maintained as isofemale lines.

#### Creation of a Bona Fide SNP Catalog for the Primary E&R Study

We generated Pool-Seq data for the three replicates of F0 from females only and for the three replicates in F70 (sex ratio ∼ 50:50). DNA extraction, barcoded library preparation, and sequencing followed standard procedures and are given in supplementary table SI 1, Supplementary Material online. We followed standard approaches for quality control, read mapping, read filtering, trimming as well as SNP calling and SNP filtering.

We used libraries with different insert sizes, which can result in false positives (Kofler et al. 2016). To account for this, we expanded the double-mapping procedure suggested in Kofler et al. (2016) and used three different mappers NovoAlign (http://novocraft.com, last accessed December, 2015), Bowtie2 (Langmead and Salzberg 2012), and BWA-MEM (Li and Durbin 2009). We filtered for biallelic SNPs outside repeat regions and removed SNPs from positions outside the 99% quantile in terms of genome-wide coverage. From this set of prefiltered SNPs, we keep only those for which the SNP frequency did not differ between all three mappers (*P* > 0.01, after False Discovery Rate [FDR] correction). We call this procedure triple-mapping. This resulted in a set of 2,560,538 high-quality SNPs. Details are given in supplementary material I, Supplementary Material online.

#### Identifying Regions under Selection in the Primary E&R

We performed Fisher’s exact tests (FET) between the ancestral F0 and the evolved F70 generation within each replicate and CMH tests (Agresti 2002) across replicates. As coverage variability (see supplementary table SI 2, Supplementary Material online) affects the power of FET and CMH tests, we used the independent hypothesis weighting (IHW) procedure (Ignatiadis et al. 2016) to weight the empirical *P* values using the mean coverage at each SNP calculated from all replicates included in any particular test, as a covariate.

To determine the list of candidate SNPs, we ran neutral forward Wright–Fisher simulations for each replicate based on replicate-specific *N*_e_ estimates (table 1) that we obtained for autosomes and the X chromosome using the poolSeq package (Taus et al. 2017) and mimicking the starting frequencies and empirical coverages at each SNP. Neutral *P* values were also submitted to the IHW procedure. Candidate SNPs from either FET or CMH tests were declared at a 1% FDR cutoff, applying a conservative nonparametric empirical FDR estimator (Strimmer 2008) using the weighted *P* values from our simulations and the weighted *P* values from our observed data.

**Table 1:** Autosomal *N*_e_ Estimates of the Primary and Secondary E&R Experiments

	Replicate *x*	Replicate *y*	Replicate *z*
**Primary E&R**	206	263	226
**Secondary E&R**	134; 144	—	216; 193; 167

Selection coefficients were determined for each SNP in each replicate on pseudo-count data (detailed in supplementary material I, Supplementary Material online) using the poolSeq package assuming a dominance coefficient of 0.5.

### The Secondary E&R Experiment

#### Experimental Population, Selection Regime, and Sequencing

Based on the primary E&R selection signature screen, we picked a candidate region on 3R (region details in supplementary fig. 1, Supplementary Material online) for further investigation. This region showed a very strong signal of positive selection in a CMH test across replicates *x*, *y*, and *z*. We used evolved flies from replicates *x* and *z* after 77 generations of evolution in the primary E&R experiment (F77) to set up a secondary E&R experiment in which the evolved flies were mixed with flies from a reconstituted ancestral population (Nouhaud et al. 2016, supplementary fig. 2, Supplementary Material online). We call this generation D0. Selection targets are expected to increase in frequency again in the secondary E&R experiment, which used the same culturing conditions as the primary E&R experiment.

Mixing proportions of ancestral and evolved populations to create D0 were chosen such that selected SNPs in our candidate region had allele frequencies falling in a deterministic range between 0.25 and 0.75 in D0: for replicate *x*, a 30:70 ratio between evolved and reconstituted ancestral flies, and for replicate *z*, a 50:50 ratio, respectively. We created two replicates for D0 for replicate *x* (*x*.1 and *x*.2), and three replicates for D0 for replicate *z* (*z*.1, *z*.2, and *z*.3). Replicates for D0 and D30 were subjected to Pool-Seq.

#### Validation of Signatures of Selection in the Secondary E&R

Selection coefficients and neutrality tests were performed exactly as described for the primary E&R experiment.

## Results

### Discovery of Candidate SNPs: Primary E&R

**Fig. 1.**
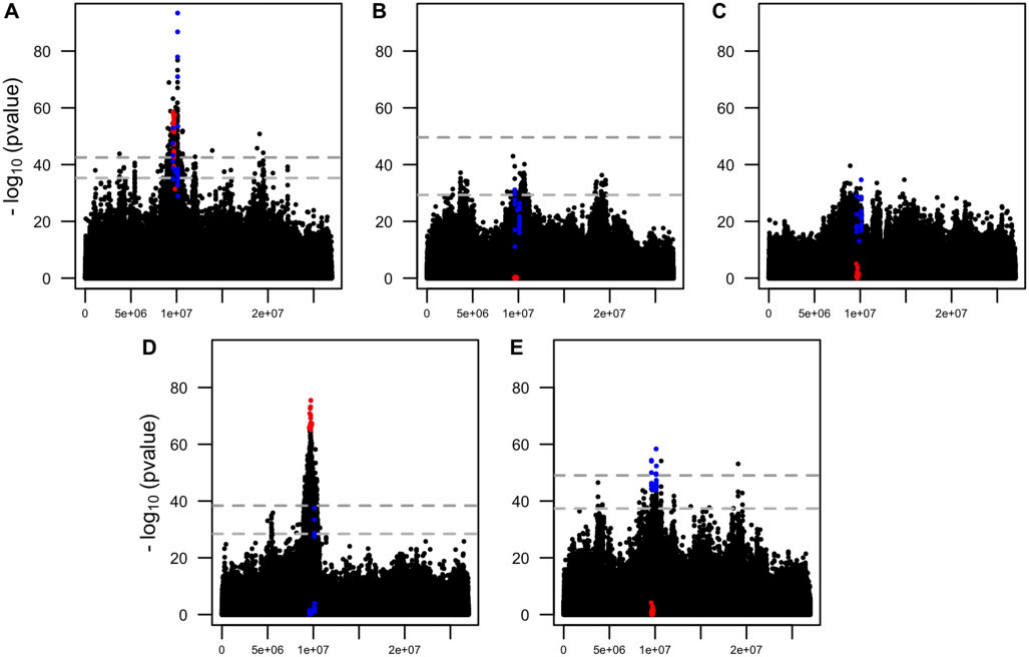
—Replicate-specific selection signatures in the primary E&R study. Manhattan plots of chromosome arm 3R displaying the negative log 10-transformed weighted *P* values of 680,937 SNPs for different statistical tests. (*A*) CMH_*x*__,__*y*__,__*z*_ (175/443), (*B*) FET_*y*_ (0/122), (*C*) FET_*z*_ (0/0), (*D*) FET_*x*_ (660/1,776), and (*E*) CMH_*y*__,__*z*_ (9/85). The number of candidates at 1%/5% empirical FDR thresholds for each test is given in parentheses. The gray dotted line shows the 1% (upper) and 5% (lower) empirical FDR thresholds of the corresponding test, computed over the autosomes from neutral simulations assuming no linkage. At the 1% empirical FDR threshold, CMH_*x*__,__*y*__,__*z*_ and FET_*x*_ identify a candidate peak region of 169 (9,042,023–10,670,451 bp, 1.628 Mb) and 660 (9,000,008–10,384,933 bp, 1.385 Mb) SNPs. The overlap between these 2 tests is 92 significant SNPs spanning 1.343 Mb (see supplementary fig. SI 1, Supplementary Material online, for a close up of this genomic region). In all panels, the top 20 SNPs from FET_*x*_ and CMH_*y*__,__*z*_ are highlighted in red and blue.

Three replicates of a *D. simulans* founder population were maintained in a constant hot environment (23 °C) for 70 nonoverlapping generations. Sequencing pools of 1,250 individuals (Pool-Seq, Kofler and Schlötterer 2014; Schlötterer et al. 2014; supplementary table SI 1, Supplementary Material online) resulted in a catalog of 2,560,538 polymorphic SNPs (see Materials and Methods, supplementary table SI 2, Supplementary Material online). We identified candidate SNPs by contrasting allele frequency changes (AFCs) between ancestral and evolved populations with a CMH test after accounting for drift using a 1% empirical FDR threshold (see Materials and Methods). Because *P* values obtained from contingency tables tests are affected by coverage, we also accounted for coverage heterogeneity among samples (56*x*–261*x*, supplementary table SI 3, Supplementary Material online) by weighting *P* values following the IHW procedure (Ignatiadis et al. 2016) (see Materials and Methods). The genome-wide analysis identified a candidate region of 1.628 Mb on chromosome arm 3R with a pronounced AFC between ancestral and evolved populations (fig. 1, top left, the full genomic analysis will be published elsewhere).

The power of the CMH test relies on the experimental replicates to detect putative targets of selection. However, its utility is limited when candidates are not shared across replicates. Analyzing this genomic region separately for each of the replicates using a FET indicated considerable heterogeneity among them: Among the SNPs with the most significant CMH *P* values across all three replicates, the top 20 SNPs in the FET of replicate *x* were only significant in replicate *x* (FET_*x*_, fig. 1, bottom left, top center, top right, red), with 16 SNPs being close to fixation. Removing replicate *x* from the CMH analysis and using only replicates *y* and *z*, we obtained a much weaker selection signature in the CMH test (CMH_*y*__,__*z*_, fig. 1, bottom right). Only 3 of the 20 most significant SNPs of this analysis (CMH_*y*__,__*z*_) were overlapping with the most significant SNPs of the analysis including *x* (CMH_*x*__,__*y*__,__*z*_). Instead, the 20 most significant SNPs of CMH_*y*__,__*z*_ changed in both replicates *y* and *z* with a mean AFC of 0.55. This AFC is less pronounced than the one observed for the significant SNPs of replicate *x* (0.96). This heterogeneity among replicates suggested that at least two distinct classes of haplotypes were selected.

**Fig. 2.**
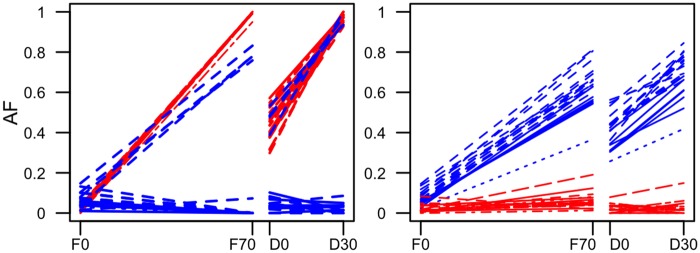
—AFCs of the 20 most significant SNPs from FET_*x*_ (red) and CMH_*y*__,__*z*_ (blue) for the primary E&R (generation F0–F70) and secondary E&R (D0–D30). Different types of lines are used to better distinguish the AFCs from each SNP. The left panel shows experiment *x* and the right panel experiment *z*. Only the first replicate from the secondary E&R is shown for each experiment, for the other replicates, see supplementary fig. SI 3, Supplementary Material online.

We further scrutinized the hypothesis of at least two distinct selected haplotypes and plotted the AFC of the two sets of top 20 SNPs in the candidate region on chromosome arm 3R (fig. 2): 20 SNPs from FET_*x*_ and 20 SNPs from the joint analysis of replicates *y* and *z*, that is, CMH_*y*__,__*z*_. The two sets of candidate SNPs displayed group-specific AFC; one set showed a pronounced AFC in replicate *x* and the other one in replicate *z*, but almost no change in the other (fig. 2 and supplementary fig. SI 1, Supplementary Material online).

### Validation of Candidate SNPs: Secondary E&R

The primary E&R study provided two sets of candidate SNPs. One set of candidates increased strongly in replicate *x* only, whereas the other set of candidates increased weakly in the two replicates *y* and *z*. To demonstrate that both sets of SNPs are associated with a selection target, we aimed to validate both selection signatures experimentally. Reasoning that fewer replicates are needed to confirm strong selection, only two diluted replicates were generated from evolved replicate *x* (*x*.1 and *x*.2), whereas three diluted replicates were generated from evolved replicate *z* (*z*.1, *z*.2, and *z*.3) which showed the weakest response in the initial E&R experiment. For both secondary E&R experiments, we added flies from a reconstituted founder population (Nouhaud et al. 2016) aiming for a starting frequency around 0.5 for the most prominent candidate SNPs (see supplementary fig. SI 2, Supplementary Material online). This starting frequency of the candidate SNPs in the secondary E&R ensures a deterministic selection response and still provides sufficient opportunity for frequency increase.

**Fig. 3.**
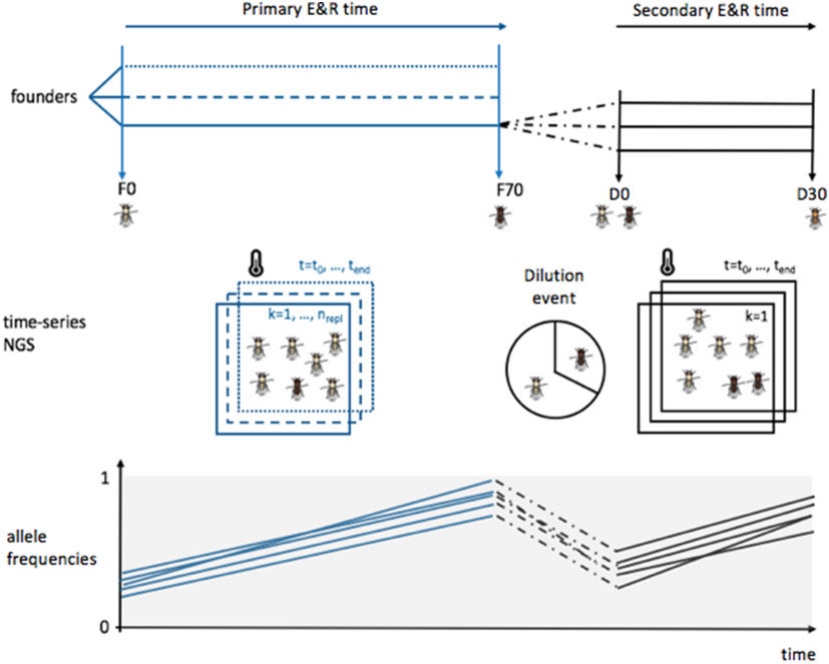
—Schematic outline of the experimental design. Three replicated populations of flies starting from the same founders evolved in parallel during 70 generations (primary E&R) and sequenced at time points *t*. The darkness of the flies symbolizes the level of adaptation to the new environment. For a given evolved replicate *k*, the evolved flies are “diluted” with ancestral genotypes and independent replicates evolving for an additional 30 generations under the identical environmental conditions as in the primary E&R (secondary E&R, indicated in black). The bottom panel indicates the replicate-specific AFCs of candidate SNPs during the experiments. In the primary E&R, the allele frequency increases (blue). By adding ancestral genotypes, the frequency of the candidate SNPs is decreased (black dotted lines). Thirty generations of the secondary E&R result in a repeated frequency increase of the candidate SNPs, confirming nonneutral evolution.

After 30 generations of evolution at the same culture conditions, we sequenced the founders (D0) and evolved replicates (D30) of the secondary E&R experiments (see fig. 3 for an overview). We contrasted the dynamics of the two groups of top candidate SNPs in each of the replicates in the primary and secondary E&R experiments over four time points (F0, F70, D0, and D30). A very pronounced frequency increase can be noted in both the primary and secondary E&R experiments in the focal replicate from which the candidates were obtained (fig. 2 and supplementary fig. SI 3, Supplementary Material online). From an average starting allele frequency of 0.52 and 0.31, the candidate SNPs reach a mean final frequency of 0.98 (*x*) and 0.73 (*z*) in the replicates of the secondary E&R. The consistent AFC in the primary and secondary E&R experiments confirms a high repeatability of selection. This suggests that the AFCs are very consistent in primary and secondary E&R experiments, and selection in the primary E&R study is a reasonable predictor of the secondary E&R.

Also, the candidate SNPs from the nonfocal replicate consistently failed to show selection signatures (fig. 2 and supplementary fig. SI 3, Supplementary Material online). The only exception are four SNPs from the candidate set of replicate *z*, which also increased in frequency in the primary and secondary E&R of replicate *x* (fig. 2 and supplementary figs. SI 3 and SI 6, Supplementary Material online). Because the AFC was less pronounced than the one of the focal candidate SNPs of replicate *x*, we conclude that these SNPs may be shared between the two alternatively selected haplotype classes.

**Fig. 4.**
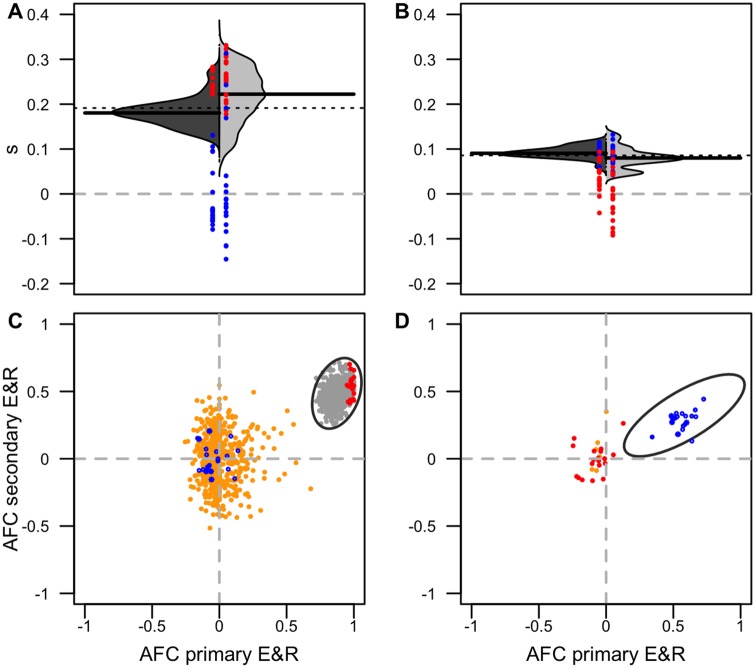
—Repeatability of selection signatures in primary and secondary E&R. (*A*, *B*) Selection coefficients (*s*) are very similar in primary and secondary E&R. Symmetrical violin plots of the selection coefficients from primary E&R (dark gray) and the first replicate of the secondary E&R experiment (light gray) for candidate SNPs (empirical FDR < 1%) in the region of interest. Black horizontal bars represent the median *s* for primary and secondary E&R. The dotted line shows the median *s* across both E&R experiments. (*A*, *C*) Experiment *x*. (*B*, *D*) Experiment *z*. The 20 most significant SNPs from FET_*x*_ (red) and CMH_*y*__,__*z*_ (blue) are shown in color across all panels. (*C*, *D*) Parallel changes in allele frequencies. Observed AFCs in the primary E&R (*x* axis) and in the first replicate of the secondary E&R (*y* axis) for candidate SNPs (empirical FDR < 1%) measured in replicate *x* (*C*) and *z* (*D*) are shown in gray, with the 20 most significant SNPs highlighted in red/blue. Because, for replicate *z*, no SNP exceeded the empirical FDR of 1% in the primary E&R, the top 20 SNPs are shown (right panel). Ellipses around the empirical focal SNPs indicate the 99% probability range. For comparison, the expected neutral AFCs for candidate SNPs are shown in orange. A single neutral simulation was performed to obtain the expected neutral AFC using the same starting frequency and coverage for the candidate SNPs as in the empirical data. All SNPs were assumed to be unlinked.

For a more complete picture, we expanded our analysis of the 20 most significant SNPs to all significant SNPs (FDR < 0.01) of the primary E&R. We jointly plotted the distribution of selection coefficients obtained from the primary and secondary E&R experiments (see Materials and Methods). Consistent with the previous analyses, all candidate SNPs had a selection coefficient larger than 0 in their focal replicate—independently of whether primary or secondary E&R experiments were analyzed (fig. 4*a* and supplementary fig. SI 4, Supplementary Material online). The inferred selection coefficients for replicate *x* are about twice as high as the ones for replicate *y*. The mean selection coefficients from the 20 candidate SNPs are 0.26 and 0.27 for diluted replicates from *x* (0.26 in the primary E&R) and 0.08, 0.09, and 0.12 for diluted replicates from *z* (0.09 in the primary E&R). As expected, the selection coefficients of the nonfocal top 20 candidate SNPs were distributed around 0.

Finally, to evaluate the influence of genetic drift, we simulated the dynamics of the significant SNPs (FDR < 0.01) in the primary E&R under neutrality and compared them with their observed dynamics (fig. 4*b* and supplementary fig. SI 5, Supplementary Material online, and Materials and Methods). Plotting the pairwise observed and simulated neutral AFC of the primary E&R against the AFC of the secondary E&R experiment, we find that the simulated data are clearly distinct from the experimental ones. The significant SNPs of the experimental data cluster together in the upper right quadrant and do not overlap with neutral simulations, showing that genetic drift cannot explain the concordant signatures of the significant SNPs. As expected, the separation of neutral and selected SNPs was clearer for the replicate *x*, where selection was stronger (fig. 4a).

### No Increased Mapping Resolution for the Selection Target

Given that the dilution reduced the frequency of the selection target, we anticipated that additional recombination events occurring during the repeated spread of the selection targets would also increase the mapping resolution. Nevertheless, we noted that the selection signature was broader in the secondary E&R experiment than in the primary one (supplementary fig. SI 6, Supplementary Material online). Hence, despite the highly repeatable selection signature of candidate SNPs, the secondary E&R experiment did not yield more confidence about the focal target of selection than the primary E&R experiment.

## Discussion

One of the undisputed advantages of experimental evolution is that the precise experimental conditions are known, which allows to impose the same selection pressure on different populations and time points in a replicated manner. Independent detection of candidate loci is widely considered strong empirical support for selection, rather than genetic drift.

In this report, we expand this concept by testing for repeatable genomic selection signatures by a simple manipulation of the evolved populations. By adding unevolved genotypes, we reduce the frequency of the selection target, which provides the opportunity to monitor a repeatable frequency increase of selected alleles in replicated populations. However, this procedure changes allele frequencies genome wide and provides the opportunity of new epistatic interactions that were not possible in the founder or the populations in the primary E&R study. Such new epistatic interactions may result in novel selection targets that were not uncovered in the primary E&R. It will be extremely challenging to distinguish between new selection targets created by epistatic interactions and the heterogeneity of polygenic adaptation (Yeaman et al. 2018). Similarly, epistatic interactions may also affect the spread of the focal selection target in E&R experiments. Nevertheless, unless epistatic interactions predominate, it should be possible to confirm selected variants by experimentally manipulating allele frequencies in the population in which a favorable variant spread. Indeed, we confirm this by demonstrating that this novel approach accurately recovers the selection signature of candidate SNPs.

Despite the mapping resolution of the primary E&R experiment could not be improved, it is striking how consistent the selection coefficients of the top candidate SNPs were replicated in the secondary E&R experiments.

Previously, experimental evolution studies exposed laboratory-evolved populations to selection regimes in the opposite direction (reverse evolution) (Teotónio and Rose 2001; Porter and Crandall 2003; Teotónio et al. 2009). The secondary E&R design introduced here also relies on already laboratory selected populations, but rather than changing the selection regime, the same selection regime is applied after manipulating the evolved population. Secondary E&R is designed to provide researchers additional confidence about selection targets by repeating a genomic selection signature in replicate populations after adding genotypes from the founder population, which reduces the frequency of selected alleles. We propose that secondary E&R experiments with unevolved genotypes provide an attractive approach to experimentally validate selection signatures. This is particularly important for either nonreplicated or small AFCs—both signatures of polygenic adaptation.

The strength of secondary E&R experiments is well illustrated in our proof of principle study, in which no single SNP passed the genome-wide significance threshold in this genomic region in the primary E&R experiment in replicate *z*. Only by combining two replicates, *y* and *z*, we identified significant candidates, which could be confirmed in the secondary E&R experiment. Thus, we demonstrated that even populations with weak selection signatures can be used to confirm the presence of selection, which could not be recognized before.

What is the conceptual advantage of secondary E&R compared with a larger primary E&R experiment? Polygenic adaptation from a founder population with high genetic redundancy typically results in heterogeneous selection responses, thus challenging the typical confirmation strategy of parallel selection responses. A good demonstration of this is provided by Barghi et al. (2019). Despite a powerful experimental design with 60 generations and 10 replicated populations, several selection targets were detected only in 1 or 2 replicates. Because the redundancy in the founder population becomes only apparent after the primary E&R experiments, it is not possible to determine the number of replicates needed to achieve parallel selection responses in a sufficiently large number of replicates. Hence, secondary E&R experiments provide a viable confirmation strategy for candidates detected in one or a few replicates only.

Secondary E&R experiments are not fast, the 30 generations of this experiment took about 14 months, but the maintenance of replicate populations does not require many resources and no experimental phenotyping is required for laboratory natural selection experiments, such as the one of this study. Secondary E&R provides therefore a very good approach to experimentally validate genomic regions experiencing selection. Mapping of causative variants could not be achieved in this pilot study and requires alternative approaches to do so. In fact, our data suggest that the mapping accuracy of secondary E&R may be lower than by adding additional primary E&R replicates—if the selection target increases in sufficiently many replicates. Consistent with this, we do not define repeatability based on the signature of an individual SNP, but based on the collective selection signature of SNPs linked to the selection target. Note, that the correlation structure of SNPs linked to the selection target obviates the need to genotype the actual selection target in a secondary E&R experiment.

Nevertheless, the dynamics of selected genomic regions are highly informative of the underlying genetic architecture of beneficial mutations. Polygenic adaptation to a novel trait optimum displays characteristic dynamics (Franssen et al. 2017), which are best detected in multiple replicates. We anticipate that the analysis of multiple replicates in secondary E&R experiments will provide an unprecedented opportunity to study replicated dynamics of selection targets in order to understand the architecture of adaptation. It is also conceivable to use this experimental setup to study the dynamics of a given selected region in an alternative selection regime.

A particularly interesting pattern could be confirmed in this study: Two different haplotype classes are carrying adaptive variants that increase fitness of the populations in a novel hot environment. It is particularly remarkable that the two groups of haplotypes seem to be mutually exclusive—we see either one or the other increasing in frequency in the primary E&R experiment. Also in the secondary E&R experiments, we see no evidence of parallel selection of both haplotype classes, but their different starting frequencies in the secondary E&R considerably decrease the opportunity for a strong frequency increase of the haplotype with the lower starting frequency. The mapping resolution is not high enough to determine whether the same gene is carrying a beneficial mutation in both haplotype classes or different genes are selected. Thus, similar to many other E&R studies, a good strategy for fine mapping is needed to answer these questions.

## Supplementary Material

Supplementary data are available at *Genome Biology and Evolution* online.

## Supplementary Material

evaa036_Supplementary_Data

## Notes

**Data deposition:** Data sets (sync format), the results of Cochran–Mantel–Haenszel and Fisher’s exact tests, the estimated selection coefficients, coverage values, and empirical and simulated polarized frequencies for both evolve and resequencing experiments in each replicate in 3R arm are available from the Dryad Digital Repository: https://doi.org/10.5061/dryad.mkkwh70vs.
