# Supplementary material for: Secondary Evolve and Resequencing: An Experimental Confirmation of Putative Selection Targets without Phenotyping
Source: Genome Biol Evol. 2020 Apr 6;12(3):151–9. doi: 10.1093/gbe/evaa036 (PMC7144549; doi:10.1093/gbe/evaa036)
Supplement: evaa036_Supplementary_Data [file evaa036_supplementary_data.zip › table1_supplement_GBE_update_format.docx]

**Table SI 1** Sequencing information.

| **Library** | Primary E&R experiment: Ancestral replicates (F0) | Primary E&R experiment: Evolved replicates (F70) and Secondary E&R experiment: Diluted ancestral (D0) and evolved replicates (D30) |
| --- | --- | --- |
| **Method for DNA isolation** | High salt extraction protocol including RNase A treatment from Miller et al. 1988 | High salt extraction protocol including RNase A treatment from Miller et al. 1988^.^ |
| **Amount of starting material (genomic DNA)** | 5µg | 1µg |
| **Fragmentation method** | Covaris S2^1^ | Covaris S2^1^ |
| **Kit** | TruSeq DNA PCR-Free Sample Prep Kit^2^ | NEBNext Ultra DNA II Library Prep Kit^3^ |
| **Size selection method** | AMPure XP beads^4^ | AMPure XP beads^4^ |
| **Insert size (bp)** | 380 | 260 |
| **Polymerase** | PCR-free | Q5 Mastermix |
| **No. of PCR cycles** | PCR-free | 4 |
| **Sequencing platform** | HiSeq 2000 | HiSeq XTEN |
| **Read length (bp)** | 2x100 | 2x150 |
|  |  |  |

**1) Covaris, Inc. Woburn, MA, USA**

**2) Illumina, San Diego, CA**

**3) New England Biolabs, Ipswich, MA**

**4) Beckman Coulter, Carlsbad, CA**
