# Supplementary material for: Secondary Evolve and Resequencing: An Experimental Confirmation of Putative Selection Targets without Phenotyping
Source: Genome Biol Evol. 2020 Apr 6;12(3):151–9. doi: 10.1093/gbe/evaa036 (PMC7144549; doi:10.1093/gbe/evaa036)
Supplement: evaa036_Supplementary_Data [file evaa036_supplementary_data.zip › table2_supplement_GBE_update_format.docx]

**Table SI 2** Candidates list from the primary E&R study.

|  | raw number of variants | | | filtered number of variants | | | | | intersection over the 3 mappers | coverage filtering  and mapping  outliers filters |
| --- | --- | --- | --- | --- | --- | --- | --- | --- | --- | --- |
|  | NovoAlign | Bowtie2 | BWA-MEM | NovoAlign | Bowtie2 | | | BWA-MEM |  |  |
| SNPs  autosomes/  X | 3988599/  636251 | 3905323/  622883 | 4010418/  640279 | 2865514/  452947 | 2737761/  431690 | | | 2876308/  454144 | 2532855/  404290 | 2225925/  334613 |
| INDELs  autosomes/  X | 404255/  85078 | 314352/  69037 | 394719/  83311 |  | |  |  |  |  |  |
