# Supplementary material for: Secondary Evolve and Resequencing: An Experimental Confirmation of Putative Selection Targets without Phenotyping
Source: Genome Biol Evol. 2020 Apr 6;12(3):151–9. doi: 10.1093/gbe/evaa036 (PMC7144549; doi:10.1093/gbe/evaa036)
Supplement: evaa036_Supplementary_Data [file evaa036_supplementary_data.zip › table3_supplement_GBE_update_format.docx]

**Table SI 3** Average coverage from sync file at SNP positions per sample for NovoAlign counts for primary (secondary) E&R.

|  |  |  |  |  | |
| --- | --- | --- | --- | --- | --- |
|  | **Primary E&R** | | **Secondary E&R** | | |
|  | F0  autosomes / X | F70  autosomes / X | D0  autosomes / X | | D30  autosomes / X |
| **Replicate x**  x.1-x.2 | 167/167 | 89/77 | 54-49/47-42 | | 67-67/59-56 |
| **Replicate y** | 256/256 | 79/68 | - | | - |
| **Replicate z**  z.1-z.2-z.3 | 185/185 | 88/74 | 58-80-37/50-66-32 | | 71-72-78/59-60-65 |
