# Supplementary figures and images for: Secondary Evolve and Resequencing: An Experimental Confirmation of Putative Selection Targets without Phenotyping
Source: Genome Biol Evol. 2020 Apr 6;12(3):151–9. doi: 10.1093/gbe/evaa036 (PMC7144549; doi:10.1093/gbe/evaa036)

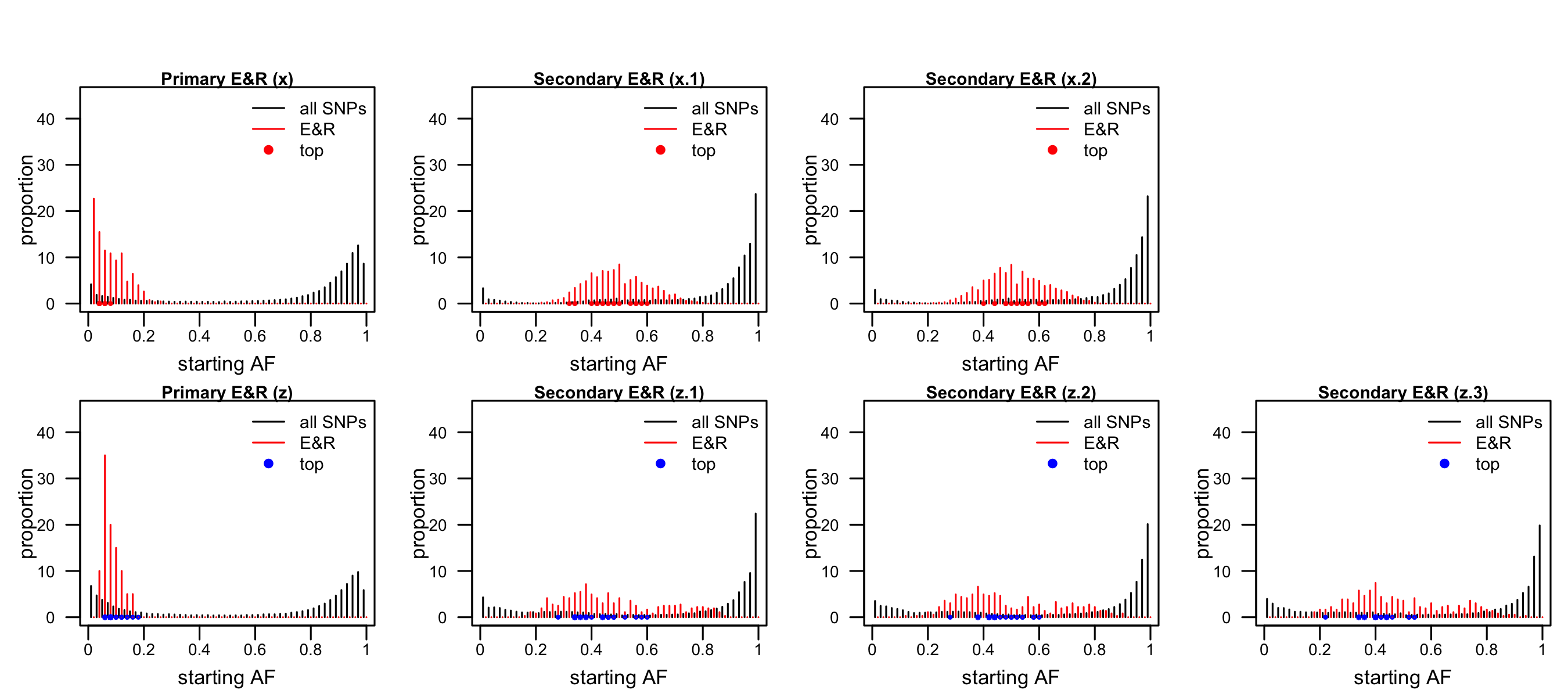

Supplement: evaa036_Supplementary_Data [file evaa036_supplementary_data.zip › FigSI2_lwz.tif]

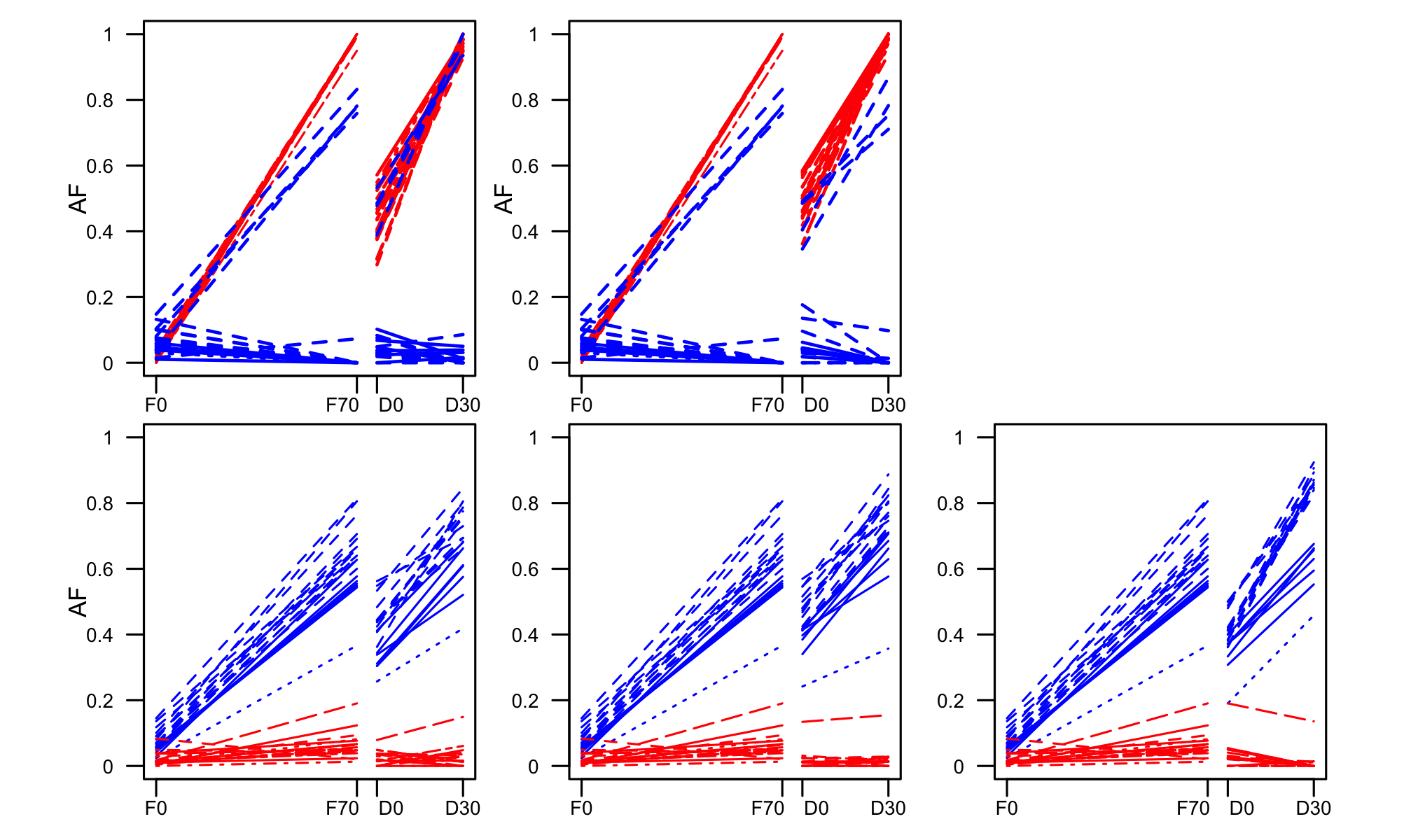

Supplement: evaa036_Supplementary_Data [file evaa036_supplementary_data.zip › FigSI3_lwz.tiff]

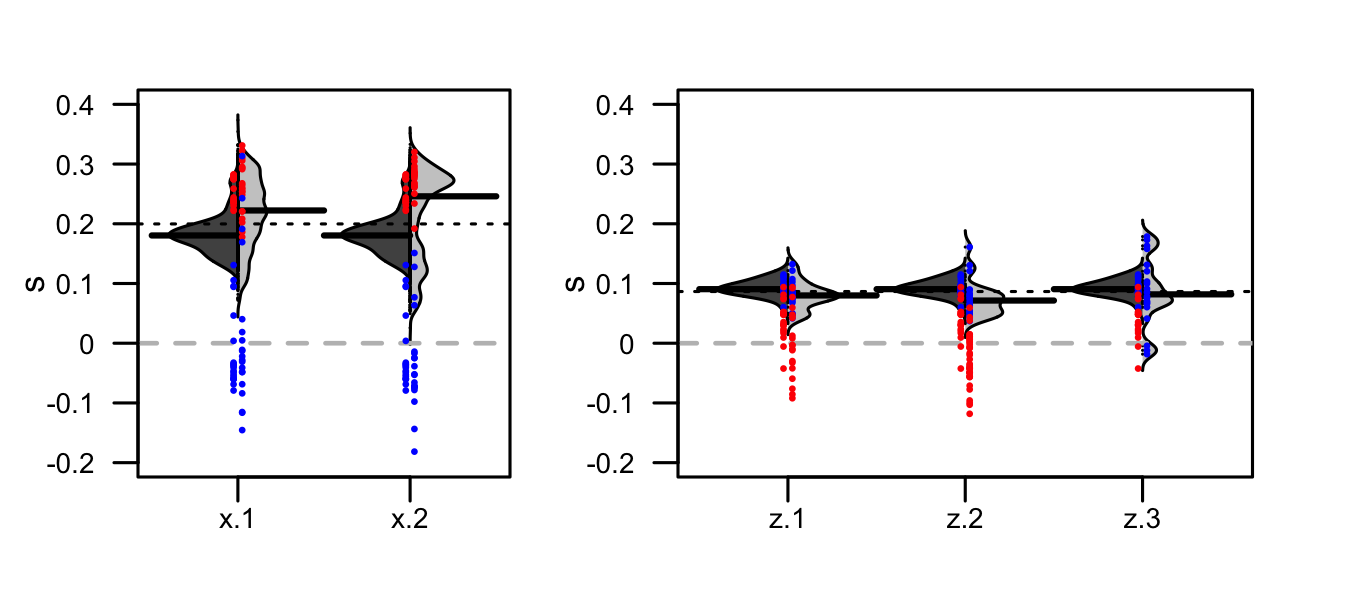

Supplement: evaa036_Supplementary_Data [file evaa036_supplementary_data.zip › FigSI4_lwz.tiff]

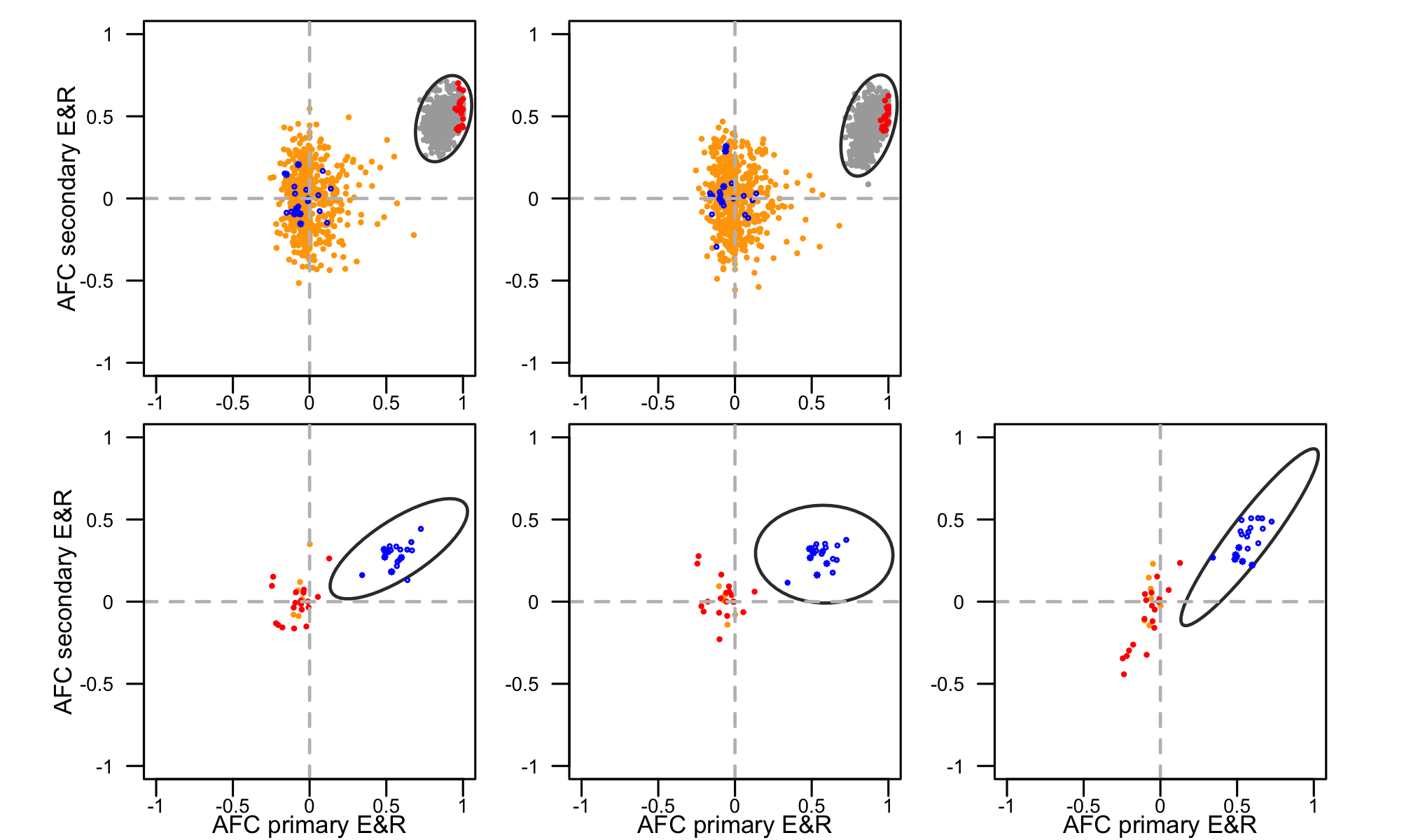

Supplement: evaa036_Supplementary_Data [file evaa036_supplementary_data.zip › FigSI5_lwz.tif]

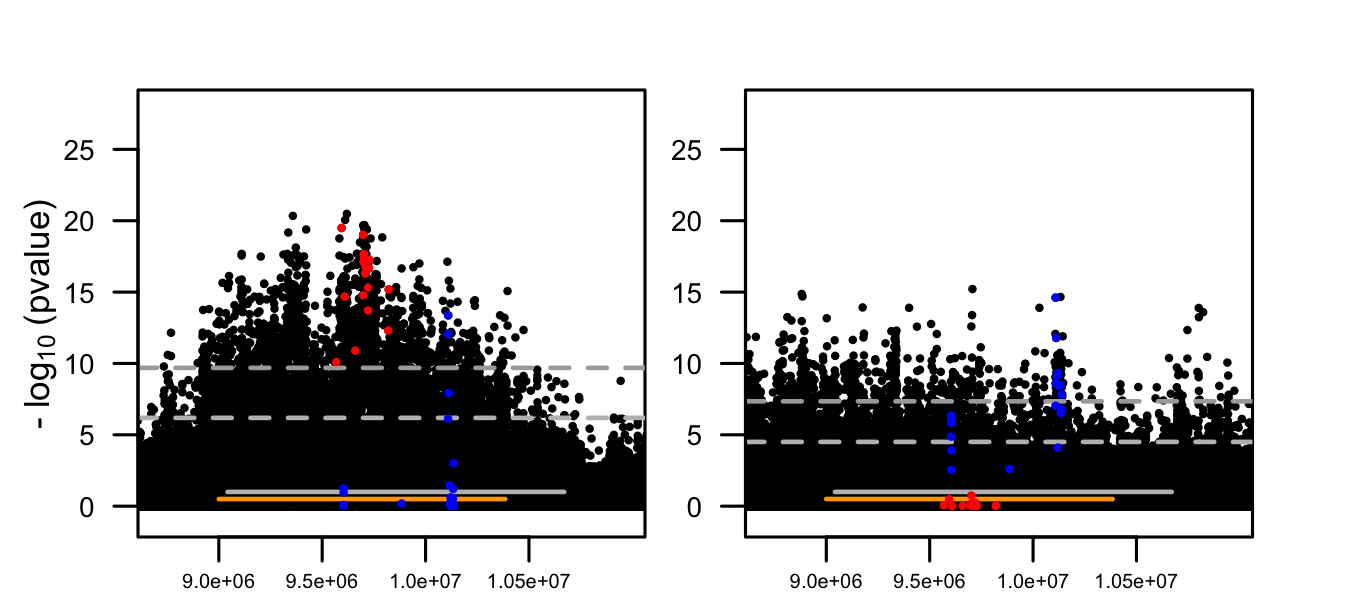

Supplement: evaa036_Supplementary_Data [file evaa036_supplementary_data.zip › FigSI6_lwz.tif]

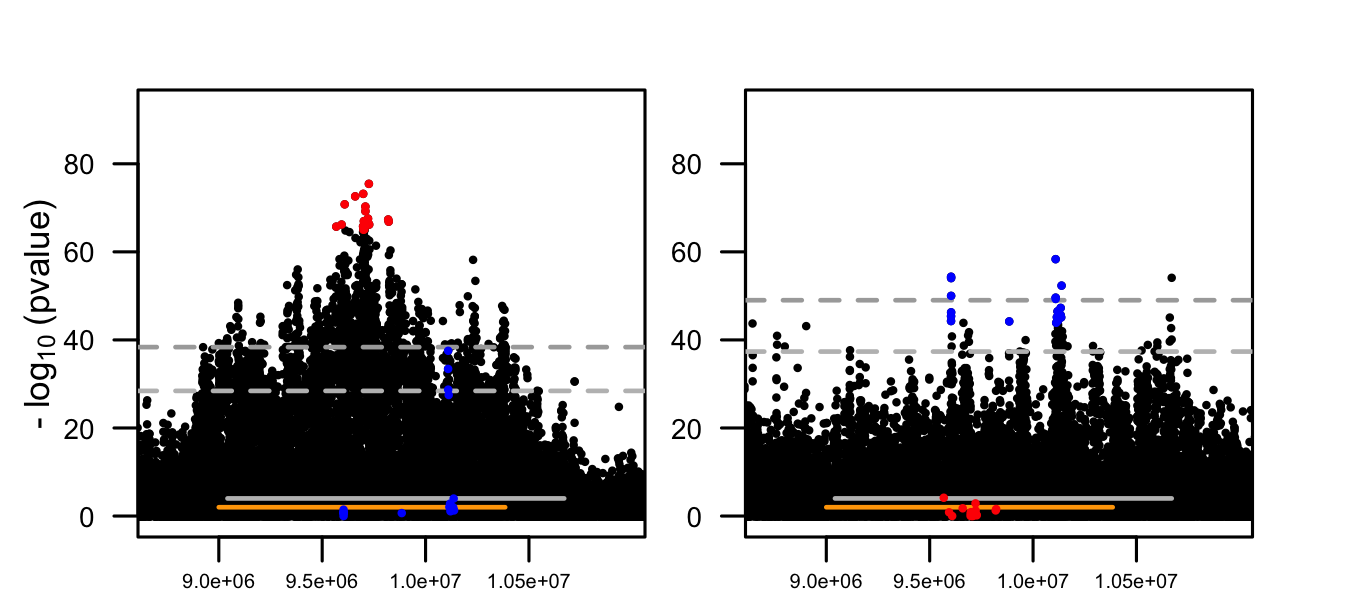

Supplement: evaa036_Supplementary_Data [file evaa036_supplementary_data.zip › FigSI1_lwz.tif]
